# The place for people in rewilding

**DOI:** 10.1111/cobi.14318

**Published:** 2024-07-01

**Authors:** Joseph Glentworth, Anna Gilchrist, Rowan Avery

**Affiliations:** ^1^ Department of Planning, Property and Environmental Management, School of Environment, Education and Development University of Manchester Manchester UK; ^2^ Department of Natural and Built Environment Sheffield Hallam University, City Campus, Howard Street Sheffield UK

**Keywords:** cultural landscapes, human–nature relationships, nonhuman autonomy, restoration, rewilding, autonomía no humana, paisajes culturales, relación humano‐naturaleza, renaturalización, restauración

## Abstract

Rewilding, although controversial, is increasingly presented as humanity's best hope of addressing the global biodiversity crisis, but it remains unclear how restoring nonhuman autonomy affects people's relationships with nature. We conceptualized 3 human–nature relationships (HNRs) that could occur when restoring nonhuman autonomy: human–nature dichotomy, human–nature compromise, and human–nature mutualism. Through 51 interviews, we then empirically tested the occurrence of these HNRs across diverse actors living and working in 2 longstanding British rewilding initiatives to better understand the place for people in rewilding. Actors’ HNRs aligned with the 3 conceptual framings, but these relationships were complex. Individuals often demonstrated multiple perspectives that transcended conventional actor categorization. The tripartite framing also revealed conflicting values across and within individuals, resulting in pluralistic HNRs. Our work adds to the theory and practice surrounding the place for people in rewilding by cautioning against a single preferred HNR when restoring nonhuman autonomy and advocating that a diversity of human interactions with nature should be integrated into the global rewilding movement.

## Abstract (es)

El lugar de las personas en la renaturalización

**Resumen:**

Aunque la renaturalización es controversial, se presenta cada vez más como la mejor esperanza para que la humanidad aborde la crisis mundial de biodiversidad, aunque todavía no está claro el efecto de la restauración de la autonomía no humana sobre las relaciones entre las personas y la naturaleza. Conceptualizamos tres relaciones humanidad‐naturaleza (RHN) que podrían ocurrir al restaurar la autonomía no humana: dicotomía, equilibrio y mutualismo, todas entre los humanos y la naturaleza. Realizamos 51 entrevistas para probar de forma empírica la ocurrencia de estas RHN con varios actores que viven y trabajan dentro de las dos iniciativas británicas de renaturalización más antiguas y así entender mejor el lugar de las personas en la renaturalización. Las HNR de los actores se alinearon con los tres marcos conceptuales, aunque estas relaciones fueron complejas. Los individuos frecuentemente mostraron tener varias perspectivas que trascendían la categoría de los actores. El marco tripartito también reveló valores conflictivos entre y en los individuos, lo que resultó en RHN pluralistas. Nuestro trabajo suma a la teoría y práctica en torno al lugar de la gente en la renaturalización con la prevención de una sola relación humanidad‐naturaleza preferida cuando se restaura la autonomía no humana y con la recomendación de que la diversidad de interacciones humanas con la naturaleza debería integrarse al movimiento mundial de renaturalización.

## INTRODUCTION

Rewilding is widely considered as an indispensable strategy in addressing the global environmental crises (Carlson et al., 2023; Carroll & Noss, 2021; Svenning, 2020), shifting conservation priorities from habitat and species protection to restoring the emergent properties and processes of ecosystems (Jepson, 2016; Perino et al., 2019). Rewilding emerged in North America (Appendix S1A) but has since been applied in different contexts, including extensively modified cultural landscapes, where its meaning has evolved and diversified (Gammon, 2018; Deary & Warren, 2017). Despite this variety, a dominant conceptual theme is restoring wildness or enhancing nonhuman autonomy (hereafter NHA) (DeSilvey & Bartolini, 2018; Wynne‐Jones et al., 2020). We consider NHA to mean that the qualities and trajectories of aspects of nonhuman nature are self‐willed and self‐sustaining (Prior & Ward, 2016; Ward & Prior, 2020). Critically, enhancing NHA requires a transformative change in human–nature relationships (HNRs) and a “paradigm shift in the coexistence of humans and nature” (Carver et al., 2021, p. 1890).

This shift is pronounced when rewilding occurs in cultural landscapes shaped by millennia of human occupation (e.g., Europe) that enmesh the natural with deep‐seated social and historical values (Linnell et al., 2015). Defining and implementing NHA in relation to the role of people however is “one of the most difficult elements of rewilding discourse to reconcile” (Martin et al., 2021, p. 7). For instance, the International Union for Conservation of Nature (IUCN) considers the long‐term aspiration of rewilding is to reduce anthropogenic intervention, whereby ecosystems are restored to a point where they require “no or minimal management” (Carver et al., 2021). Alternative perspectives posit that people are part of ecosystems and that rewilding should reconfigure humanity's role as “destructive” “hyper‐ecosystem engineers” (Jepson, 2022, p. 4) and promote landscapes that are “co‐habituated” and “co‐shaped” by both humans and nonhumans (Prior & Ward, 2016, p. 134). Such contrasting positions demonstrate the extent of disagreement about the appropriate HNRs needed to successfully implement rewilding.

Our contribution is 2‐fold. First, we respond to a recognized gap in the conceptualizations underpinning rewilding concerning how HNRs materialize when restoring NHA. We used literature to develop a novel framework that characterized 3 different representations of HNRs that can emerge as rewilding restores NHA. Our second contribution then applies this tripartite framework across these themes to examine the HNRs of a diverse range of actors in 2 long‐standing rewilding projects. This responds to the empirical knowledge gap relating to the practice of rewilding, which concerns the need for research that focuses on “the value pluralism associated with rewilding and current land use…and attitudes towards and support for rewilding” (Massenberg et al., 2023, p. 49). To frame the empirical analysis, we used 4 key themes dominant in the existing European rewilding literature. These themes include how restoring NHA to cultural landscapes challenges traditional land‐based livelihoods and rural economies (Tănăsescu, 2017; Wynne‐Jones et al., 2018), confronts the cultural aesthetics of landscapes scenery (Drenthen, 2018; Prior & Brady, 2017), questions biodiversity management (Jepson, 2016) and reconsiders traditional approaches to natural resource management (Pereira & Navarro, 2015).

## PLURALITY OF HNRs IN REWILDING

Nonhuman and nature's autonomy are concepts that have gained increasing attention in and outside the rewilding literature (Heyd, 2005; Thomas, 2016). Assigning autonomy to nonhumans has been rejected by some on the grounds of Kantian ethics (Kant, 1785; Korsgaard, 2018) (see Appendix S1B). Others have challenged these ideas, calling for a “naturalized” view of autonomy (Thomas, 2016, p. 87) and demanding respect for nature's autonomy in relation to people (Hettinger, 2005). Nonhuman agency is another related concept occasionally used interchangeably with autonomy (Vasile, 2022), but it is less well‐established in rewilding literature. We therefore used the following definition of NHA: “an ethos of relinquishing direct human management of wild organisms or ecological processes, and one that foregrounds the self‐directed actions of nonhumans” (Ward & Prior, 2020, p. 104). This conceptualization has featured prominently in recent rewilding studies (e.g., Dempsey, 2021; Schulte to Bühne et al., 2021; Thomas, 2021; Wynne Jones et al., 2020).

Interest in HNRs extends across diverse disciplines, giving rise to a variety of conceptual orientations (Appendix S1C). This has been synthesized by IPBES (2022), which presented 4 life frames that help categorize HNRs: “living from,” “living with,” “living in,” and “living as” nature (Pascual et al., 2023). Critical to our work is the recognition by IPBES that HNRs are not absolute or homogeneous but instead complex and pluralistic (O'Neill et al., 2008).

Conceptualizing HNRs within rewilding is challenging because most existing framings present HNRs in the context of static or declining biodiversity and regard humans as responsible for controlling the reversal of this state through management interventions. This is captured by Folke et al. (2021), who frame different, evolving HNRs over time and predict harmonious HNRs emerging from increased awareness of worsening environmental degradation. In contrast, rewilding aims to alter the socioecological baseline by increasing NHA, thereby relinquishing human control (to some degree) (Ward, 2019), which we see as challenging existing notions of HNRs. The IPBES recognizes this knowledge gap, reporting that “more study is needed on how values are affected by conservation interventions, for example, …ecological restoration and shifting socio‐ecological baselines” (IPBES, 2022, p. 99). We therefore drew largely from the rewilding literature to conceptualize the place for people in rewilding.

### Rewilding as human–nature dichotomy

Jørgensen (2015) argues that rewilding upholds a dualistic dichotomy between humans and nature. This view is evident in early literature and discourse on rewilding, where the goal was to restore wilderness. One early extreme advocate of rewilding famously stated, “humanity is the cancer of nature […] the optimum human population of Earth is zero” (Foreman et al., 1992, p. 73). In this interpretation, restoring NHA equates to absolute independence of long‐term human influence.

The dichotomous view of rewilding often accepts that initial anthropogenic action, such as restoration and reintroduction, may be necessary to heal the “wounds […] caused by abusive land uses” (Soulé & Noss, 1998, p. 24). However, this framing aspires to rewilding that is entirely self‐sustaining and devoid of human influence (Carver et al., 2021), aligning with noninterventionist ideals (Katz, 1992). This is grounded in overwhelming evidence that human population growth and associated impacts on ecosystems have had a profound degrading effect on local and global ecology (Foley et al., 2005). However, longstanding criticism of “fortress conservation” has been applied to rewilding (Pettorelli et al., 2018, p. 1118), highlighting the problems of promoting undisturbed nature and the associated displacement of local communities (Cronon, 1995; Holmes et al., 2020).

### Rewilding as human–nature compromise

Literature suggests a dichotomous HNR is not inevitable. Local communities can retain or adapt income sources or employment in rewilding projects (Recio et al., 2020; Tănăsescu, 2017). Similarly, cultural heritage and identity are being integrated into rewilding projects (DeSilvey & Bartolini, 2018). Some researchers have interpreted this integration of people as “taming” (Martin et al., 2021) or “domesticating” (Thomas, 2021) rewilding, with cultural or practical realities forcing a concession to a true wilderness ethic (Leduc & von Essen, 2019). Nonetheless, this framing offers pragmatism by restoring degrees of NHA in heavily modified or populated landscapes. The compromise HNRs that emerge can therefore be understood as a byproduct of complex trade‐offs between NHA and anthropogenic influence.

### Rewilding as human–nature mutualism

Arguably, this compromise perspective still underpins an environmental ethic where “a policy of human/nature apartheid would be best” (Hettinger, 2005, p. 88). Instead, rewilding can be framed as a way of restoring mutually beneficial relationships between humans and nature (Tănăsescu, 2019). In ecology, mutualism describes a cobeneficial interaction between 2 different organisms (Linnell et al., 2015). Under this framing, the autonomy of both become enmeshed, with human practices shaped by nature while simultaneously strengthening NHA:

Restoring to the rural landscape wolves that might eat our sheep forces us to change our grazing practices, adds to nature's influence over our lives, and lessens our control of the situation; thus it likely increases the autonomy of local nature in relation to humanity (Hettinger, 2005, p. 93).

This conceptualization of rewilding highlights the importance of ongoing anthropogenic interaction with nonhumans, provided this does not control the trajectory of the natural system or its components. Mutualism therefore raises an important distinction between harmonious interaction and controlling intervention (DeMello, 2012).

## METHODS

The above framework offers a conceptualization of the types of HNRs that may emerge as NHA is increased through rewilding. Yet, a significant gap exists between rewilding theory and practice. Indeed, some argue that rewilding is dominated by academic voices (Gammon, 2018) and does not appreciate on‐the‐ground contexts (e.g., Linnell et al., 2015). Thus, we used the tripartite framework to examine the HNRs emerging from 2 rewilding projects in cultural landscapes in England.

### Case study overview

We applied a dual case study method (Stake, 2013), based on 2 long‐standing projects documented elsewhere in the literature, which have been increasing NHA by restoring natural processes (Thomas, 2021, 2022). Wild Ennerdale (WE) in northern England is among the earliest examples of upland rewilding (Convery & Dutson, 2012) and is promoted as an exemplar initiative by Rewilding Britain (Appendix S1D). The project covers 4300 ha previously managed for hill farming and non‐native forestry. Under rewilding, the landscape is transitioning to upland cattle grazing and passive vegetation management. Long‐term aspirations are to transition to deciduous upland woodlands.

The Avalon Marshes (AM) cover 1500 ha of southwestern English lowlands, comprising low‐lying land that before drainage and sea defenses was a mixture of salt marsh, reed swamp, and raised bog. Peat soils historically supported agriculture and peat extraction, which intensified through the 20th century. Following peat industry decline in the 1990s, hydrological management was used to flood the excavated fields, creating wetland reedbeds that reflected prehistoric land cover. Numerous species have naturally recolonized the area (see Thomas, 2021).

Like most UK projects, neither enterprise defines their work as rewilding (Sandom & Wynne‐Jones, 2019) because the term is considered highly controversial in landscapes that have strong cultural histories (Drenthen, 2018). Nonetheless, both specifically aim to increase NHA (Perino et al., 2019) by improving connectivity and dispersal, enhancing trophic complexity, and increasing stochastic disturbances (Table 1).

**TABLE 1:** Context of the 2 case studies used to examine the place for people in rewilding.

	Wild Ennerdale (WE)	Avalon Marshes (AM)
Location	Lake District National Park (Northwest England)	Somerset Levels (Southwest England)
Historic context	Archaeological evidence demonstrates human habitation between 2000 and 800 BC. Over 500 years of traditional sheep farming practices, now generally perceived as embodying the area's cultural history and traditions. Non‐native conifers, predominantly Sitka Spruce (*Picea sitchensis*), planted for timber in the 1920s, although timber industry has declined in the last 3 decades. Viability of upland farming declined in late 20th century, as extensive agriculture struggled to compete with lowland agricultural intensification in the latter half of the century and a policy shifted away from payments for numbers of livestock (see Mansfield [2014] for a more detailed account).	Archaeological evidence demonstrates continuous human habitation from as early as 3838 BC. In prehistory, covered by large wetlands, reclaimed during Roman era and used for agriculture. Over 200 years of peat extraction, now generally perceived as embodying the area's cultural history and traditions. Viability of peat industry declined in late 20th century in connection with national environmental campaigns against the global extractive peat industry, and emergence of alternative horticultural products.
Background of rewilding initiative	Wild Ennerdale project evolved over 1990s and officially established in 2003. Rewilding led by partnership of statutory bodies (Natural England, Forestry Commission), private companies (United Utilities), and NGOs (National Trust).	Land given to conservation in 1990s following decline of peat industry, then flooded to create wetlands. Rewilding led by Avalon Marshes Partnership composed of statutory bodies (Environment Agency, Historic England, Natural England, Somerset County Council) and NGOs (Hawk and Owl Trust, RSPB, Somerset Wildlife Trust, South West Heritage Trust).
Rewilding focused on connectivity and dispersal	Large‐scale area within Lake District National Park identified as rewilding zone. Extensive tree planting to promote woodland habitat connectivity including over 100,000 native broadleaf trees and 10,000 Juniper trees (*Juniperus communis*). Two kilometers of forest roads have been removed out of the forest road network and are being allowed to vegetate. Active restoration of wetland habitats to promote connectivity.	Large‐scale parcels of wetland reedbed and open water areas restored amounting to over 900 ha, created in strategic network of adjacent land ownership to enhance wetland habitat connectivity. Retained wet woodland parcels interspersed through wetlands under low‐intensity traditional management (e.g., selective thinning) Continual, ongoing land purchases by conservation bodies to increase area of conservation. Promotion of natural recolonization, particularly flagship avian species, including locally extinct, e.g., common crane (*Grus grus*) and novel species, e.g., great white egret (*Ardea alba*).
Rewilding focused on trophic complexity	Over 1500 ha of the valley is accessible to 3 herds of extensive grazing Galloway cattle (both within and outside of the forest area). Promoting an evolving mix of land uses and blurring of traditional boundaries between forestry, farming, and wild land. Reintroduction of species, including marsh fritillary butterfly (*Euphydryas aurinia*).	Naturalistic grazing of distinct areas including reedbed margins and fields by old‐breed cattle (Ruby Red Devon and Highland) and Exmoor ponies. Water management using drains that can be pumped in both directions creates a heterogeneous mosaic of habitat types, with a system of weirs downstream allowing penning of water to hold sufficient supply in summer but protect from winter flooding.
Rewilding focused on stochastic disturbances	Reduction of upland sheep grazing. Passively managed areas with no active intervention where natural disturbances such as flooding are actively given space to shape the landscape. Altered infrastructure along the river to promote natural processes (e.g., replaced a forest road bridge with a single span bridleway bridge to kick start natural gravel flow).	Patchwork landscape of diverse reedbed, open water, woodland, and grassland areas with varying degrees of passive management or active interventions applied.

*Note*: Inventory of the main rewilding interventions applied per site was qualitatively characterized by the authors based on the 3 ecological rewilding components characterized by Perino et al. (2019).

### Data collection

During 2018, we conducted 51 semistructured interviews across the 2 projects (WE = 23; AM = 28), aiming to examine people's attitudes and relationships with nature as a result of restoring NHA (interview guide in Appendix S2). Sampling targeted 6 broad actor categories separated into 12 more detailed categories (Table 2). Although actor groupings helped inform data collection, most interviewees self‐identified across multiple categories (Appendix S3). Sampling intended to capture maximum diversity in perspectives rather than to achieve statistical representativeness of actor type. Nonetheless, we used frequencies throughout the results to express how common each perspective was among interviewees (hereon actors). Data collection continued at both sites until no new substantive material emerged (Miles & Huberman, 1994). Ethical approval for this research was obtained from the University of Manchester prior to fieldwork.

**TABLE 2:** Categories of actors connected to Avalon Marshes and Wild Ennerdale interviewed to examine the place for people in rewilding.^a^

Broad	Specific	Wild Ennerdale (WE)	Avalon Marshes (AM)
Residents	Residents (long term)^b^	11	20
	Residents (short term)^b^	4	6
Farmers	Farming	6	5
	Farming (undertaking conservation)	5	3
	Farming (family background)	6	6
Peat cutters	Peat cutting (mechanized)	N/A	4
	Peat cutting (hand dug)	N/A	3
	Peat cutting (family background)	N/A	7
Nature conservationists (C)	Conservation professionals	6	6
	Conservation volunteers	9	8
Recreationists (R)	Recreationist, site user, nature watcher	13	20
Heritage specialists (H)	Heritage specialists and professional	3	2

^a^ In most cases, interviewees were classified in multiple different actor categories (see Appendix S3 for actor categorization of each actor).

^b^ Definitions: long term, ≥5 years; short term, ≤5 years.

### Data analyses

The interviews were transcribed and coded using an iterative, multilevel thematic approach (Braun & Clarke, 2012). Four dominant themes emerged from literature on rewilding in cultural landscapes and were used to frame a process of deductive coding. The 4 themes were categorized as biodiversity and ecological function (Jepson, 2016), natural resource use and ecosystem services (Pereira & Navarro, 2015), rural economies and livelihoods (Recio et al., 2020), and cultural‐heritage and landscape aesthetics (Drenthen, 2009; Gammon, 2018). We used inductive coding to identify unifying subthemes; people's perceptions on the impacts of rewilding were grouped around statements, such as “past culture is obscured or alienated by wildness” or “communities can form around rewilding initiatives.” We used a third round of deductive coding to determine how these themes related to the 3 types of HNRs (Appendix S3).

## RESULTS

Table 3 summarizes the percentage of interviewees that aligned with different subthemes for each case study (28 in AM, 23 in WE); the frequency is detailed in the text.

**TABLE 3:** Empirical examples of the subthemes aligned to the 3 types of human–nature relationships derived from interviews with actors connected to Avalon Marshes and Wild Ennerdale.

	Human–nature dichotomy		Human–nature compromise		Human–nature mutualism	
Theme	Subtheme	Actor % and type	Subtheme	Actor % and type	Subtheme	Actor % and type
Biodiversity and ecological function	Enforced physical separation from nature.	AM: 7% F, L, P, R WE: 26% C, F, L, R	Management interventions are necessary to conserve and enhance biodiversity.	AM: 82% AA WE: 65% AA	Increasing NHA promotes unanticipated positive outcomes for biodiversity.	AM: 36% C, F, L, R WE: 48% C, F, L, R
	Perceived decline in biodiversity associated with rewilding.	AM: 14% C, F, L, R WE: 30% C, F, L			There is acceptance of novel hybrid natures.	AM: 25% C, F, L, R WE: 13% C, R
	Removing past land uses can increase biodiversity and ecosystem functioning.	AM: 68% AA WE: 22% C, R				
Natural resource use and ecosystem services	Rewilding does not deliver tangible goods to society.	AM: 25% C, F, L, P, R WE: 22% C, F, H, L	A mix of land use types delivering different benefits is desirable.	AM: 86% C, F, L, P, R WE: 78% AA	Ecosystem services are a byproduct of functioning ecosystems.	AM: 14% C, L, R WE: 35% C, F, L, R
			Food production can occur on wildland.	AM: 25% C, F, L, P, R WE: 35% C, F, L, R		
Economy and livelihoods	Rewilding leads to a decline of traditional rural communities and loss of rural income.	AM: 39% AA WE: 52% AA	Rewilding and economically productive land uses can coexist through compromises.	AM: 57% AA WE: 74% AA	Rewilding can support declining rural economies.	AM: 32% C, F, L, P, R WE: 30% C, F, L, R
Cultural heritage and landscape aesthetics	Rewilding debases working heritage.	AM: 25% F, H, L, P, R WE: 35% AA	Heritage features and traces can be retained in rewilding.	AM: 68% AA WE: 70% AA	Rewilding can be framed as a cultural initiative.	AM: 43% C, F, L, R WE: 48% C, F, H, L, R
	Past culture is obscured or alienated by wildness.	AM: 39% AA WE: 39% AA			Communities can form around wild places.	AM: 54% AA WE: 65% AA
	Rewilding creates spectacular people‐free aesthetics.	AM: 64% C, F, L, P, R WE: 48% C, H, L, R				

Abbreviations: AA, all actor types; C, nature conservationists; F, farmers; H, heritage specialists; L, local residents; P, peat cutters (Avalon Marshes only); R, recreationists.

### Biodiversity and ecological function

Restoring NHA resulted in distinct perspectives toward biodiversity. 6 subthemes were revealed that aligned with different HNRs, 3 to dichotomy, 1 to compromise, and 2 to mutualism (Table 3).

Under dichotomous HNRs, 6 actors at WE, including farmers, local residents, and conservationists, associated rewilding as a process of physically separating people from nature. One farmer stated “they have closed the gate and locked it” about a farm that was being passively managed (WE10). At AM, this theme was less dominant; 2 actors reported a feeling of separation resulting from rewilding, but this was also linked to cultural detachment from the cessation of peat working.

Slightly more actors (AM = 4; WE = 7) perceived a decline in biodiversity associated with the rewilding initiatives, especially where passive management was occurring. This perception was held mostly by farmers but also a few conservationists and recreationists. Farmers at WE noted that the area was dominated by “gorse, bracken, and Molinia” (WE8) and natural regeneration “was primarily non‐native spruce” (WE20). There was concern that non‐native invasive species were not appropriate and were spreading because of decreased intervention. Four actors, all involved in farming, expressed similar anxieties regarding reduced interventions at AM, citing reductions in culling of predators (such as corvids and mustelids) as a driver of perceived biodiversity decline.

In contrast, recreationists and conservationists at AM commented that biodiversity increased because of reduced human intervention, such as peat harvesting (AM = 19). One conservationist went further and expressed a view that human–nature dichotomy is very positive for biodiversity: “Chernobyl, that's true rewilding, because we've left it the hell alone, […] it's amazing for wildlife” (AM21). Positive perspectives of dichotomy were less frequent in WE (WE = 5), although the biodiversity benefits of removing some past intensive farming practices in key locations were observed: “stepping back from the landscape completely” can have a “really positive” impact on biodiversity (WE14).

Compromise HNRs were also evident relating to biodiversity and ecological function. Both projects committed to increasing NHA; however, most actors at AM (AM = 23) believed that the rewilded landscape “absolutely needs management and monitoring” (AM1). This centered around preserving valued species or habitats through traditional conservation management, which would be seen by some as not adhering to rewilding principles. This view was also held widely within WE (WE = 15), but conservation professionals frequently outlined that the initiative tried to avoid specific species or habitat targets. They stated, for example, such a prescriptive approach was against “the ethos of the project” (WE1). However, these conservationists stressed that rewilding required intervention at varying levels across the area, such as tree planting and vegetation management. The need for intervention was frequently raised in relation to the Galloway cattle (*Bos taurus*), viewed in WE as engineers of naturalistic grazing (Vera, 2000). Actors working at WE believed welfare provided by the grazier (e.g., supplementary feeding) helped reduce criticism from farmers.

Two mutualistic themes were evident relating to biodiversity. One, mentioned at both sites (AM = 10; WE = 11), highlighted positive unintended outcomes that the projects had on wildlife that were directly linked to the open‐ended management promoted by rewilding. Five recreationists and all conservationists at AM mentioned the spontaneous return of long‐extirpated breeding birds, such as great egret (*Area alba*) and black‐crowned night heron (*Nycticorax nycticorax*), that had occurred since the start of the project because of increasing NHA. One actor said, “Bittern wasn't known here, and great white egret was […] a dream” (AM10). The open‐ended ecological benefits of providing more space for the River Liza “to create new niche habitats” (WE1) by removing restrictive infrastructure were celebrated by 4 actors working in conservation at WE. These same actors spoke positively about “working with the river” rather than against it to enhance biodiversity.

For some actors at both sites (AM = 7; WE = 3) (particularly professional conservationists), rewilding did not mean restoring a past baseline but helping create novel “future nature” (WE22). At AM, conservationists disavowed restoration to historic baselines and offered future visions of hybrid landscapes as alternatives, which were interwoven with some degree of anthropogenic management (e.g., to maintain reedbeds). At WE, however, a greater emphasis was placed on taking risks so that “land managers are not in complete control of the changes that are occurring; something to be celebrated and learnt from” (WE1). At WE, although 6 interviewees highlighted that the non‐native forest should be removed or phased out, 2 conservationists and one recreationist noted the ecological benefits of spruce for supporting the native population of red squirrel (*Sciurus vulgaris*). Indeed, WE22 highlighted the importance of thinking beyond native versus non‐native categories: “…in some cases it is really more important to have structural diversity rather than simply considering a species based on its nativeness” (WE22). In this sense, a hybrid form of wild nature was promoted at WE that embraced the interconnections between human and nonhuman processes and histories.

## NATURAL RESOURCE USE AND ECOSYSTEM SERVICES

Four subthemes were identified relating to natural resources and ecosystem services—one each aligning to dichotomy and mutualism HNRs and 2 to compromise (Table 3).

The dichotomy HNR related to rewilding initiatives not delivering tangible benefits, particularly food production, which farmers at both sites perceived as supporting national self‐sufficiency (AM = 7; WE = 5). As AM8 stated, “what if this country got short of food […] what support do you think you'd get from the public then, for deliberately turning all this land into wildlife conservation?” Farmers involved in extensive hill farming through to intensive modern grazing valued their productive relationship with the land. This was framed as a close, positive HNR, which they did not believe was replicated with rewilding. Such perspectives of positive productive value were highly contested among conservationists (especially at WE), who argued these practices were often environmentally destructive. Three farmers at WE saw traditional hill farming as balancing low‐intensity production with effective nature conservation while expressing that rewilding undervalued the nature and resources that resulted from traditional farming. In this sense, rewilding was seen to reinforce wider drivers of change that were decoupling people and communities from nature‐based ways of life.

Two compromise perspectives were identified relating to natural resource use. Forty‐two actors (AM = 24; WE = 18) stated that mixed‐use landscapes were more desirable for the community and wider society than homogenous land uses. This was evident across diverse actor types, including farmers who believed some land should be devoted to biodiversity (AM9 and WE15); conservationists who believed land should be prioritized for different ecosystem services, including flood protection, carbon storage, and food production (WE3, WE17, and WE20); and local residents and recreationists who enjoyed a mixed landscape to walk in (AM11, AM15, and WE5). All AM conservationists highlighted that agricultural productivity could “still be part of it” (AM24). This reflected a second subtheme that explicitly linked food production to wild areas (e.g., a recreationist highlighted that farmers can play an important role in influencing herbivores in the absence of carnivores [WE23]). This was seen as a compromise to ecosystems containing large carnivores, a type of rewilding currently contested in the United Kingdom.

One mutualistic theme emerged relating to natural resource use and ecosystem services. Twelve actors (AM = 4; WE = 8) stated that restoring ecosystem functionality delivered natural resource benefits to people and nature. In WE and AM, conservationists viewed rewilding as diversifying ecosystem service provision and that this land should “still be seen as providing an important service to people” (WE22), in contrast with some farmers’ perspectives on food production. Giving greater capacity for the river to shape the valley was a critical component of this:

The River Liza is […] telling us a story of how an upland river system should work […] The more freedom you give it the more it can serve a function in terms of flood mitigation […] it is helping to mitigate against flooding and some of those things can be translated to other valleys (WE1).

In AM, conservationists and some recreationists particularly focused on natural flood management as a cobeneficial ecosystem service. In this sense, rewilding was positioned as benefiting both people and nature in a range of different ways.

## ECONOMY AND LIVELIHOODS

Three economy and livelihood subthemes emerged at both sites, one each corresponding to dichotomy, compromise, and mutualism HNRs. Although broader socioeconomic processes have also influenced local changes, rewilding was often perceived as a driver, both positively and negatively (Table 3).

The dichotomy theme was expressed by approximately half of all actors (AM = 11; WE = 12), particularly long‐term residents with links to traditional land uses, who associated rewilding with local economic decline. This highlighted the perceived difficulties in monetizing rewilding in ways that benefit local communities. Actor AM3 worried that “this community is going to go,” noting a loss of income associated with rewilding, except for nature reserve car parking fees that they believed only benefited conservation bodies. Similar concerns were raised at WE, with a former farmer (WE18) stating “they have made the place wilder, but the local village doesn't get anything from that.” Long‐term residents at both sites also stated the limited prospects of employment linked to rewilding as local extractive industries were being replaced by nature conservation organizations, often perceived as outsiders. This contrasts with views held by all conservation professionals at both sites that rewilding increased local visitors and touristic spend, supporting the local community.

Actors at both sites (AM = 16; WE = 17), including recreationists, conservationists, and farmers, believed that through compromise, rewilding could support some economic productivity by “looking at how we can work a bit differently” (WE1). Farmers often perceived increased production as an ongoing objective pursued by successive generations that was now being restricted by rewilding. There were parts of both project areas, where farmers were paid to manage land for biodiversity, including in formally designated sites where management followed strict regulatory specifications. One farmer viewed this as a compromise, lamenting that their fields were “not a farmer's farm … you can't drive [food production]” (AM26), reflecting reduced agency for farmers to improve agricultural productivity within rewilding. Three farmers surrounding the WE initiative spoke of receiving payments to reduce their stocking densities within specific parcels of land, which was viewed as compromising the desired agricultural productivity of their land or “income foregone” (WE10). As such, farming within specific areas of AM and WE was seen to facilitate (or maintain) both economic productivity and NHA, but this meant each was compromised. Importantly, for 3 farmers and one local resident at WE, there was no clear long‐term vision to balance these different land‐use types.

The dichotomy and compromise views around economy and livelihoods were negative about rewilding. There were also positive perspectives of how both initiatives could support livelihoods, creating mutualistic HNRs in some locations of the sites (AM = 9; WE = 7). In WE, one farmer described how their practice had shifted from “hard‐bred productive continental cattle” to exclusively extensive “low intervention” naturalistic grazing (WE16). The same actor described their relationship with nature as positively evolving rather than compromising their aspirations: “production is less a consideration […] and the environment is more of a consideration… this part of the partnership changed my type of farming […] and my whole outlook on life” (WE16). Other forms of nature‐based economies were linked to rewilding, particularly by conservationists, including touristic spending in village shops and hospitality, although these claims were not clearly evidenced. At AM, high‐skilled conservation jobs were created locally, including habitat restoration and partnerships working across various conservation and heritage bodies. In contrast, the WE partnership primarily drew from organizations already working in the area; only one post was specifically designated to WE. At both sites, mutualistic perspectives on the economy and livelihoods were largely (although not exclusively) confined to conservationists. Other actors questioned whether tourism contributed enough to offset the losses incurred from rewilding and highlighted that conservation jobs were not targeted at village communities.

## CULTURAL HERITAGE AND LANDSCAPE AESTHETICS

Actors perceived a rich cultural history in both projects and were largely united by notions that heritage was embedded in these landscapes. Six divergent subthemes emerged—3 that aligned with human–nature dichotomy, one with compromise, and 2 with mutualism (Table 3).

Approximately one third of actors (AM = 7; WE = 8), mostly long‐term residents connected with land‐based industries, believed rewilding represented an erasure of their communities’ working history: “they're forgetting about what our older generations used to do, for their livelihood” (AM3). Individuals described a decline in local industries affecting place and identity, including reduced community activities and lost historic structures and artifacts. These emotions suggested that a way of life and traditional skillset had been debased by rewilding: “they're letting the history disappear and those lives disappear” (WE10).

Over one third of actors (AM = 11; WE = 9), including long‐term residents, highlighted the decline of traditional culture because of rewilding led to feelings of alienation from the landscape. This narrative was infused with bitterness directed at agents of rewilding who were perceived as spearheading the decline of heritage values:

The people that organize it, need to actually get their facts right first, and say ‘well actually it wasn't just about the birds and it's not about the greenery; it's about what was here before’ (AM4).

Rewilding's aesthetic acceptability varied spatially for many residents, for example, Actor WE7 highlighted how rewilding farther away from Ennerdale village promoted “wilderness qualities of nature,” but criticized “a really untidy landscape directly on the community's doorstep.” Critically, however, 29 actors (AM = 18; WE = 11) were positive about dichotomous HNRs in relation to aesthetics, highlighting that rewilding offered spectacular people‐free landscapes, for example, some recreationists likened empty spruce forests at WE to wilder ecosystems in North America.

A dominant theme (AM = 19; WE = 16) was a compromise perspective between rewilding and historical conservation, although these took distinctive approaches. In WE, acceptable limits were defined to balance NHA against protecting and celebrating the heritage features of the valley:

We came up with a series of thresholds whereby certain features would be preserved […] and other features would be passed over to nature […] often self‐will is a compromise between these different things (WE1).

Actors at AM also identified archaeological features that were “scientifically important to conserve [in their present state]” (AM21). By contrast with WE, however, experimental archaeologists have reconstructed Neolithic buildings at the Avalon Marshes Centre, which some actors suggested could be replicated for the peat industry to reduce a sense of alienation from the landscape.

Actors also commented on the compromise of NHA in relation to aesthetic acceptability, including vegetation management at AM for public access. At WE, the taming of wildness was seen as a concession to prevent alienation or perceptions of neglect because “people don't like the idea that we have just left the land abandoned, […] we never use that word” (WE1).

Two mutualism HNRs were evident relating to cultural heritage. Approximately half of actors (AM = 12; WE = 11) suggested rewilding was being framed as a positive cultural shift:

People seem fascinated because they haven't seen or heard of anything so obvious and big […] the ethos behind the project […] it's so different […] I take pride in that happening here (WE7).

Conservationists at both sites emphasized their intention that wildlands were inclusive to the community and were “very keen on empowerment, on helping people come together” (AM24). Both projects provided opportunities for nature‐based social activities (e.g., nature watching, experimental archaeology, ecological surveys, or conservation volunteering), creating communities and “new friendship circles” (AM17). These opportunities enhanced stronger place connections, reinforced by time spent in the landscape: “I'm out here from sunset on New Year's Eve, out throughout the year” (AM1). Some actors connected this sense of community pride and stewardship to a deeper sense of belonging to nature: “Nature changes here as it should […] you can form a relationship with the area just by being in it […] but a sense of stewardship too” (WE6).

Approximately 60% of all actors (AM = 15; WE = 15), particularly conservationists and recreationists, spoke of positive communal relationships that also benefited wildlife. At AM, local residents described detailed knowledge of and connection with the landscape acquired through walking and conservation management or monitoring activities and a greater sense of community because of this. The importance of this nature connection for human well‐being was explicitly recognized: “there are people who have been emotionally, and mentally saved […] by working [here]” (AM20).

## PLURALISTIC PERSPECTIVES ON HNRs AND REWILDING

Our empirical results revealed that actors responded very differently to increasing NHA through rewilding, with HNRs not aligned simply to actor categories. Significant complexity of attitudes occurred even within individuals (Table 4). Across 51 actors, only 2 gave interviews where every statement only aligned with a single HNR, whereas 30 gave perspectives that aligned with all 3 HNRs. This HNR pluralism was unevenly distributed across the subthemes, with more actors demonstrating plural HNRs in cultural heritage and landscape aesthetics and biodiversity and ecological function themes than for natural resources and ecosystem services or rural communities and livelihoods (Table 4). This suggests that some aspects of rewilding, notably cultural heritage and biodiversity, may result in more pluralistic views within actors or vary depending on the specific spatial context within rewilding sites.

**TABLE 4:** Number of actors demonstrating the extent of pluralistic human–nature relationships (HNRs) during interviews about the place for people in rewilding in Avalon Marshes and Wild Ennerdale.

	Number of HNRs (dichotomy, compromise, or mutualism) held by individuals				Actors holding multiple HNRs	
Themes	0 HNR^a^	1 HNRs	2 HNRs	All 3 HNRs	*n*	%
Across all themes	0	2	19	30	49	96.1
Biodiversity and ecological function	3	16	16	16	32	62.7
Natural resources and ecosystem services	3	28	20	0	20	39.2
Rural communities and livelihoods	5	23	19	4	23	45.1
Cultural heritage and landscape aesthetics	1	11	20	19	39	76.5

^a^ All actors mapped onto at least one HNR (dichotomy, compromise, and mutualism) but not within every theme. Zero HNR therefore indicates the number of actors that did not issue statements related to the theme.

Our results suggested that the pluralism held by individuals may have been influenced by 2 factors. One related to where and how rewilding was being applied within the sites. For instance, over one third of all actors (AM = 11; WE = 8) specifically mentioned areas where rewilding practices were more appropriate than others within the project. Three local residents at WE were supportive of the project in the upper valley for instance, but thought it had “gone too far” when a former farm was being passively managed near the village because “it was too close to the community” (WE5), triggering negative dichotomous views. Likewise, at AM, 4 actors held blanket views on the appropriateness of rewilding in the area, but a much greater number held nuanced perspectives about balancing land uses in specific spatial units across the landscape.

A second factor influencing value pluralism related to internal conflicts that actors expressed regarding how rewilding went against their own values for these changing cultural landscapes:

I like the sheep […] they know the landscape so well it's almost like they have a belonging there […] but then we need more trees and wilder areas too; that's something that I don't know how to get around, because you can't have both and make it work all the time (WE7).

This sense of internal conflict was mirrored in other actor responses. Actor AM3 lamented that rewilding obscured the family's long history of peat extraction and the community around it, forcing a dichotomous separation between this actor's heritage and the nature now growing over it. Conversely, this actor also enjoyed a mutualistic relationship with the new nature in the reserves, valuing the feelings of peace and security it gave on days when the actor's children were there for bicycle rides. Sixteen actors (AM = 11; WE = 5) expressed statements that showed internal conflicts, particularly residents and recreationists.

## DISCUSSION

Although increasing NHA is seen as one of the few unifying principles of rewilding, to date there has been little exploration of what implications this has for people's relationship with nature (IPBES, 2022; Massenberg et al., 2023). Our work offers a contribution in 3 main areas.

First, we conceptualized and empirically tested 3 ways in which rewilding can alter HNRs in cultural landscapes. Although existing literature has highlighted how rewilding can drive human–nature dichotomy (Jørgensen, 2015), we exposed this as multifaceted, spanning 8 different themes. Some, related to economic livelihoods and cultural heritage, are widely recognized, highlighting physical exclusion from landscapes, such as restricted access or inability to work in certain areas (Mikołajczak et al., 2022), or the narrative disconnect through debasing histories and declining communities (Drenthen, 2018; Wynne‐Jones et al., 2018). Conversely, around half of all actors perceived dichotomy positively, valorizing perceived increases in ecological function and people‐free aesthetics. This finding is notable because literature on rewilding in cultural landscapes usually reports actors’ experiences of dichotomy very negatively (DeSilvey & Bartolini, 2018; Jørgensen, 2015; Tănăsescu, 2017; Ward, 2019). Our findings, however, showed in some areas of the projects, many local actors aligned with the original position of early rewilding advocates in relation to human presence, which advocated acceptance “so long as ecological considerations came first” (Foreman, 1998, p. 543). These experiences of human–nature dichotomy seem distinctive from existing analysis of HNRs because of who is benefiting. Historically, advocates of wilderness conservation propounded that people‐free protected areas, as well as protecting nature, provided sociocultural benefits for visitors to the landscape (e.g., Muir, 1897). These perspectives align with living from nature values, but can also be critiqued for devaluing HNRs in local communities. Although, in contemporary biocentric framings such as “living with nature” (Pascual et al., 2023), dichotomous HNRs resulting from protected area management are still linked to ecological benefits, the sociocultural implications for local communities are almost universally presented as negative (e.g., see IPBES, 2022). Importantly, we found that alongside negative experiences, human–nature dichotomy resulting from rewilding was also perceived positively by various local actors, including residents, farmers, peat cutters, and conservationists. In this way, we contend that dichotomy can create HNRs that align with living as nature life‐frames and that physical separation from parts of the landscape does not need to create an ontological divide between humans and nature.

More common, particularly in European literature, is the assertion that rewilding requires compromise, balancing the goal of wildness against the pragmatic constraints of human demands (e.g., Leduc & Von Essen, 2019). We demonstrated that these perspectives were common across actor groups and identified 5 key themes related to compromise. Importantly, although this compromise is often framed negatively as restricting rewilding ambitions (e.g., Carver, 2014; Leduc & Von Essen, 2019), many people living and working around WE and AM viewed compromise positively (Table 3). Human–nature compromise spans multiple IPBES life frames, balancing economic livelihoods (living from nature) and cultural heritage (living in nature). Our findings emphasize, however, the concessionary emotions that can arise from striving to meet this balance.

Our results also reveal that mutualistic relationships were evident across 6 themes (Table 3); although dominant among conservation professionals, they were also held by residents, recreationists, and farmers. This framing of human–nature mutualism aligns closely with IPBES’ life‐frame “living as nature” and echoes Folke et al.’s (2021) people and nature concept, both of which emphasize intertwined autonomies of humans and nonhumans. However, our conceptualization promotes the explicit importance of increasing NHA for humans to exist as nature—a crucial aspect of rewilding (Prior & Ward, 2016), often absent in other frameworks.

As such, although our findings echo concerns that rewilding disconnects communities from their surrounding landscapes (Jørgensen, 2015; Wynne Jones et al., 2018), it also highlights that rewilding can foster a sense of belonging and attachment among local community members toward these landscapes, with all actor types recognizing that communities can form around wild places. New opportunities for connecting with nature can occur and have a profound positive effect on people's lives. Rather than harkening back to a pristine people‐free nature, rewilding can be seen to form new layers within cultural landscapes (Roe & Taylor, 2014), for example, enhanced well‐being resulting from time spent in wilder nature. Although some theoretical literature purports that rewilding creates new cultural landscapes (Gammon, 2019), we provide empirical evidence from local people directly affected by projects.

In summary, although some authors assert an ideal HNR like “people are nature” (Rigolot, 2021, p. 1758), we caution against imposing a singular preferred option on human subjects and align with IPBES in recognizing the innate occurrence of pluralistic HNRs. Thus, rewilding may aspire toward dichotomous HNRs that reflect people‐free wilderness ethics, balance ecological and social needs through compromise, or restore more mutualistic HNRs akin to cultural landscape models (Roe & Taylor, 2014). Rather than suggesting that rewilding needs to denounce wilderness preservation to avoid criticisms of colonialization (cf. Ward, 2019), our results suggest open‐minded, people‐centered engagement could be used to explore multiple pathways for rewilding, including some people‐free areas that may have widespread support.

Our second contribution relates to the pluralistic HNRs found within actor groups, within individuals, and across space. Existing literature often characterizes rewilding perspectives by actor type (van der Zanden et al., 2018; zu Ermgassen et al., 2018), based on preconceived categorizations, e.g., that rewilding projects inspire conservationists (Tokarski & Gammon, 2016). Although some of our findings align with these stereotypical views, there are clear examples where the opposite was true. Some farmers outlined how rewilding increased their connection with the land, whereas some conservationists were conflicted with how they should approach increasing NHA. Our findings emphasize that breaking down actor stereotypes will be important for rewilding to succeed, to create opportunities to bridge shared values, priorities, and beliefs (Breyne et al., 2021). Additionally, our results revealed that individuals held pluralistic perspectives toward rewilding (Table 4), countering the binary for‐and‐against arguments presented in literature (Tokarski & Gammon, 2016). Although work on HNRs has increasingly demonstrated value pluralism (Pascual et al., 2023), our research contributes to filling a knowledge gap of HNRs as ecosystems are restored (IPBES, 2022). A narrow view of rewilding as simply removing (Jørgensen, 2015) or reconnecting people with the natural world (Monbiot, 2013) fails to capture the complexity of emotions felt by all but 2 of 51 actors. Our findings suggest this value pluralism emerges for multiple reasons, including the spatial heterogeneity of rewilding within projects. We found that rewilding was spatially diverse and context dependent even within the study sites, with different levels of human intervention and access triggering various emotions, experiences, and resulting HNRs. Further research is needed that spatially analyzes rewilding practices and the diversity of HNRs that emerge over time.

Such an individualistic framing between humans and specific components of nature, within actor groups, within individuals, and across space, has important implications for the concept of HNRs, which, through its own terminology, reinforces the perception of “one nature,” from which humans are separate (Bell‐Williams et al., 2021). Based on our evidence, we advocate a move toward using the term *human–nonhuman relationships* (H–NHRs) to reflect this complexity. This language merely acknowledges humans as a separate species, rather than an entity disconnected from nature. Importantly, this is more likely to facilitate a greater recognition of “humans are nature” (Rigolot, 2021) or “living as nature” (IPBES, 2022), which, although not necessarily better or preferred HNRs, are poorly represented in academic research and valuation studies (Pascual et al., 2023).

We recognize we examined rewilding in England, considered by some as distinct from rewilding elsewhere (Thomas, 2021), but our case studies were chosen because of their rich cultural landscapes, implying potential synergies with cultural landscapes elsewhere. Although further research is needed to examine whether our H–NHRs translate to different contexts, we suggest our findings may provide important lessons for the global practice of rewilding, which is our third and final contribution.

Negative perceptions of rewilding often arise due to initiatives being imposed on unwilling participants (von Essen & Allen, 2019). We suggest that this occurs because of a failure to understand the pluralism of H–NHRs emerging from rewilding projects, which could be overcome by early consultation that maps out the diversity of H–NHRs in cultural landscapes. Rather than relying solely on expert knowledge that predetermines views of NHA, rewilding could adopt a people‐centered approach, incorporating multiple H–NHRs and drawing on community perspectives and traditional ecological knowledge (Klein & Arts, 2021; Marland, 2020). This approach could provide an ethical framework for integrating human needs with biodiversity enhancement (Frei et al., 2020), aiming for sociocultural outcomes that are as dynamic, heterogeneous, and complex as the ecological ambitions of rewilding (Perino et al., 2019). We found that diversity of H–NHRs often reflected spatial heterogeneity. For example, the IUCN rewilding principles promote core areas, corridors, and coexistence zones (Carver et al., 2021). Although it is tempting to assume that core areas, marked by minimal human intervention, may foster dichotomous H–NHRs, corridors intersecting varied land uses may encourage compromise H–NHRs, and coexistence areas with low‐intensity human use may promote mutualistic H–NHRs—these assumptions will only hold in some projects and may vary across actors.

This vision of increased NHA resulting in simultaneous, multiple realities of H–NHRs challenges much of the existing rewilding literature. Within existing frameworks, rewilding is often presented as a linear relationship where the ultimate goal of rewilding—and the end of the linear spectrum—is people‐free wilderness (e.g., the wilderness continuum [Carver et al., 2021]). Dichotomous relationships are the ideal, and any form of rewilding that brings people into these landscapes is a compromise to this wilderness state. This is not only contentious because of colonial undertones (Ward, 2019) and the broader implications for H–NHRs (Jørgensen, 2015), it is also, we contend, impractical and unsustainable. In the Anthropocene, where 9 billion humans need to make space for nonhumans, rewilding needs to move beyond assumptions of one type of optimal H–NHR for it to become a legitimate tool to address the climate and biodiversity crises. Instead, a diverse range of possible wilder futures exist, each with its own configurations of H–NHRs, which can be identified and embraced through people‐centered engagement.

## Supporting information

Appendix S1: Explanatory Notes

Appendix S2: Supplementary Material

Appendix S3: Supplementary Material
