# Supplementary material for: The place for people in rewilding
Source: Conserv Biol. 2024 Jul 1;38(6):e14318. doi: 10.1111/cobi.14318 (PMC11588981; doi:10.1111/cobi.14318)
Supplement: Supplementary file 1 — Appendix S1: Explanatory Notes [file COBI-38-e14318-s003.docx]

**Appendix S1: Explanatory Notes**

1. Rewilding emerged in North America, enhancing conditions for carnivores by connecting and restoring core areas of people-free ‘wilderness’^[[1]](#footnote-1)^ (Soulé and Noss, 1998). Defined by advocates as vast landscapes “without roads, dams, motorized vehicles, powerlines, overflights, or other artefacts of civilization” where ecological processes “that represent four billion years of Earth wisdom” could prevail (Foreman et al., 1992, p.3-4). Conceptualisations of wilderness vary from purely socially constructed notions of wilderness to physical geography definitions usually emphasising non-human features within the landscape. For a full account of this and the difference between North American rewilding using wilderness (as defined above) and European practices of rewilding underpinned by 'wildness', see Ward (2019). For a more detailed account of the issues related to wilderness see Cronon (1995).
2. The idea that animals and other non-humans can be autonomous is controversial for some, as it is sometimes reserved only for persons with cognitive capacities (Thomas, 2016 for a detailed discussion). We take no particular stance on this debate. Instead, we refer to autonomy in the way that it is presented within rewilding literature as outlined by Prior and Ward (2016) focusing on interactions between and within species and ecosystem components.
3. It should be noted that we adopt the terminology of HNRs because of its established position in academic discourse. We recognise however, that for some, this language may seem to reinforce a nature-human dualism. As such, although the term ‘nature’, is used, we are referring to all component parts of the other than human world but are not positing humans as being implicitly separate from or part of this definition (see also Bell-Williams et al., 2021).
4. Rewilding Britain are a non-governmental organisation (NGO) that support rewilding projects across England, Scotland and Wales.

1. [↑](#footnote-ref-1)
