# Supplementary material for: The place for people in rewilding
Source: Conserv Biol. 2024 Jul 1;38(6):e14318. doi: 10.1111/cobi.14318 (PMC11588981; doi:10.1111/cobi.14318)
Supplement: Supplementary file 2 — Appendix S2: Supplementary Material [file COBI-38-e14318-s002.docx]

**Appendix S2 Supplementary Material**

**Interview Guide**

The below guide was used to direct semi-structured interviews around the relevant research objectives. Interviews were mostly undertaken in one-to-one sessions. However, the interviews were open ended and allowed participants to direct the focus around key areas that were important to them within these broader topic areas. This approach enabled the interviewer to structure the interview and keep participants on track, whilst allowing the participants to share their own stories, observations, beliefs, and perceptions. The below was adapted during the fieldwork for different actors and sites but the same overarching themes remained consistent.

**Introduction**

Interviewer introduces the research and references to previously signed consent form and participant information sheet highlighting ethics and anonymity information.

**Thematic area of questions (adapted based on this overarching framing)**

1. Can you describe your background/relationship with this landscape (AM/WE)?
2. What areas of the landscape (AM/WE) do you prefer and why?
3. Do you feel a personal connection to any parts of the landscape (AM/WE)?
4. Do you think the landscape (AM/WE) has changed over time and if so, in what way?
5. How do you think the rewilding initiative (AM/WE) has influenced people and nature?
6. Do you think that anything is lost or gained when rewilding a landscape like (AM/WE)?
7. Do you think the community have a sense of ownership over the landscape (AM/WE)?
8. Do you think visitors feel connected to the landscape (AM/WE)?
9. How would you describe the story of the landscape (AM/WE)?
